# Supplementary material for: Design and Characterization of Myristoylated and Non-Myristoylated Peptides Effective against Candida spp. Clinical Isolates
Source: Int J Mol Sci. 2022 Feb 16;23(4):2164. doi: 10.3390/ijms23042164 (PMC8875392; doi:10.3390/ijms23042164)
Supplement: Supplementary file 1 [file ijms-23-02164-s001.zip › ijms-1592527-supplementary.pdf]

Supporting Information

Design and Characterization of Myristoylated and Non-Myristoylated Peptides Effective against *Candida* spp. Clinical Isolates

Francesca Bugli [1,2,†], Federica Massaro [3,†], Francesco Buonocore [3], Paolo Roberto Saraceni [3], Stefano Borocci [3,4], Francesca Ceccacci [5], Cecilia Bombelli [5], Maura Di Vito [1], Rosalba Marchitiello [1], Melinda Mariotti [1], Riccardo Torelli [2], Maurizio Sanguinetti [1,2,*] and Fernando Porcelli [3,*]

1. Dipartimento di Scienze Biotecnologiche di Base, Cliniche Intensivologiche e Perioperatorie, Università Cattolica del Sacro Cuore, 00168 Rome, Italy; francesca.bugli@unicatt.it (F.B.); wdivit@gmail.com (M.D.V.); rosalba.marchitiello01@unicatt.it (R.M.); melinda.mariotti@unicatt.it (M.M.)
2. Dipartimento di Scienze di Laboratorio e Infettivologiche, Fondazione Policlinico Universitario A, Gemelli IRCCS, 00168 Rome, Italy; riccardo.torelli@policlinicogemelli.it
3. Department for Innovation in Biological, Agrofood and Forest Systems, University of Tuscia, 01100 Viterbo, Italy; federica.massaro@studenti.unitus.it (F.M.); fbuono@unitus.it (F.B.); paoloroberto33@gmail.com (P.R.S.); borocci@unitus.it (S.B.)
4. CNR—Institute for Biological Systems, Area Della Ricerca di Roma 1, SP35d 9, 00010 Montelibretti, Italy
5. CNR—Institute For Biological Systems, Sede Secondaria di Roma-Meccanismi di Reazione, c/o Università La Sapienza, 00185 Rome, Italy; francesca.ceccacci@cnr.it (F.C.); cecilia.bombelli@cnr.it (C.B.)

* Correspondence: maurizio.sanguinetti@unicatt.it (M.S.); porcelli@unitus.it (F.P.); Tel.: +39-063-015-4218 (M.S.); +39-076-135-7041 (F.P.)

† These authors contributed equally to this work.

Table of contents:

CMC determination — Figure S1 and Figure S2

Stern Volmer modified plot — Figure S3 and Figure S4

Time evolution of secondary structure in water and TFE/water — Figure S5

Time evolution of secondary structure in POPC and POPC/POPG — Figure S6

Average density profiles across lipid bilayer — Figure S7 and figure S8

Snapshot of MD simulations at 1 µs — Figure S9

Table S1: MIC values (mg/L) of different myristoylated and non-myristoylated peptides against different isolates of *C. albicans*, *C. glabrata, C. parapsilosis, C. tropicalis* and *C. Auris.*

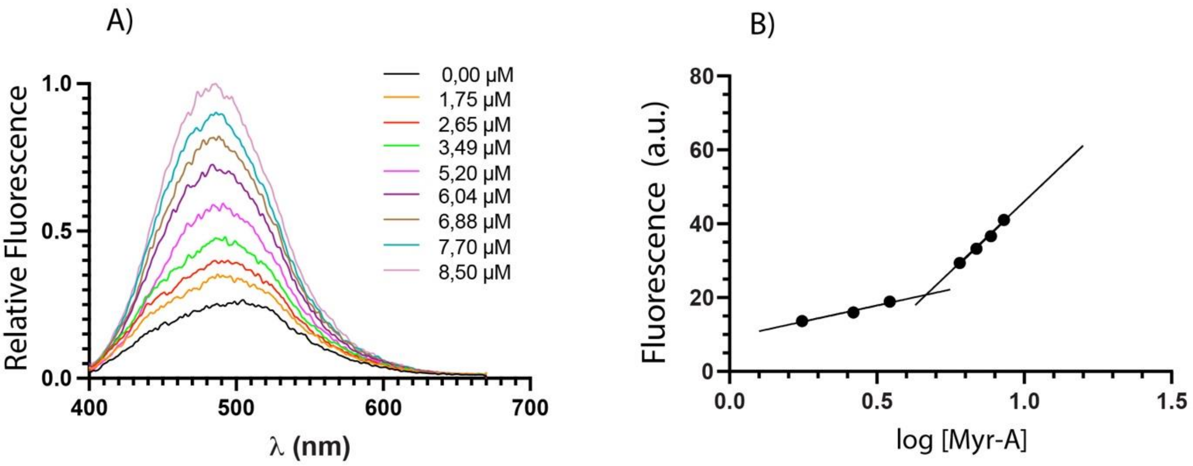

Figure S1. Determination of CMC. A) ANS fluorescence spectra of peptide Myr-A at increasing concentration. B) The plot of ANS fluorescence at 465 nm vs. logarithm of lipopeptide concentration. The intersection of the lines indicates the CMC.

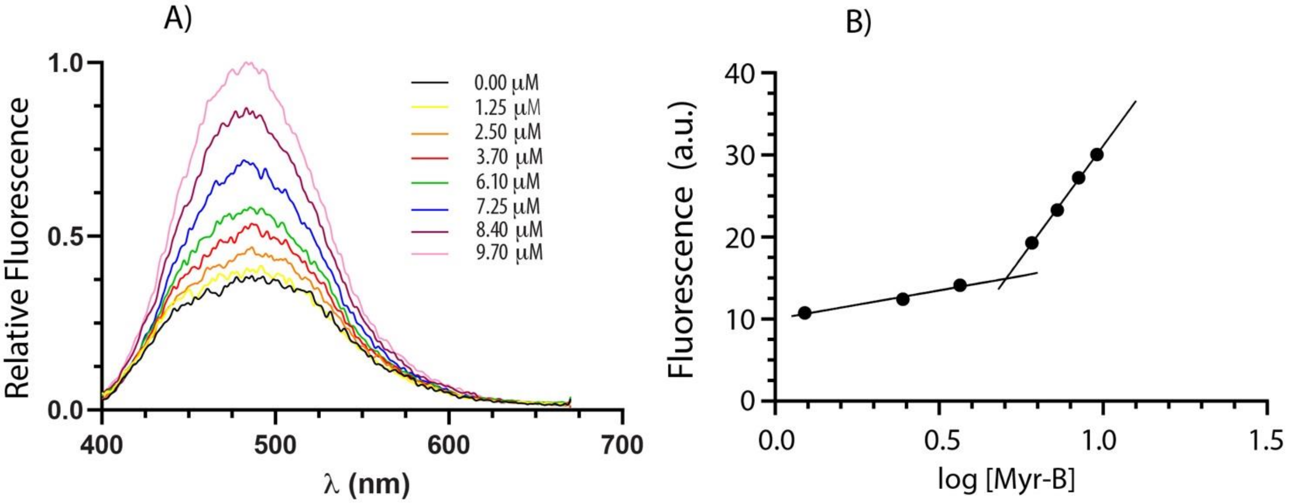

Figure S2. Determination of CMC. A) ANS fluorescence spectra of peptide Myr-B at increasing concentration. B) The plot of ANS fluorescence at 465 nm vs. logarithm of lipopeptide concentration. The intersection of the lines indicates the CMC.

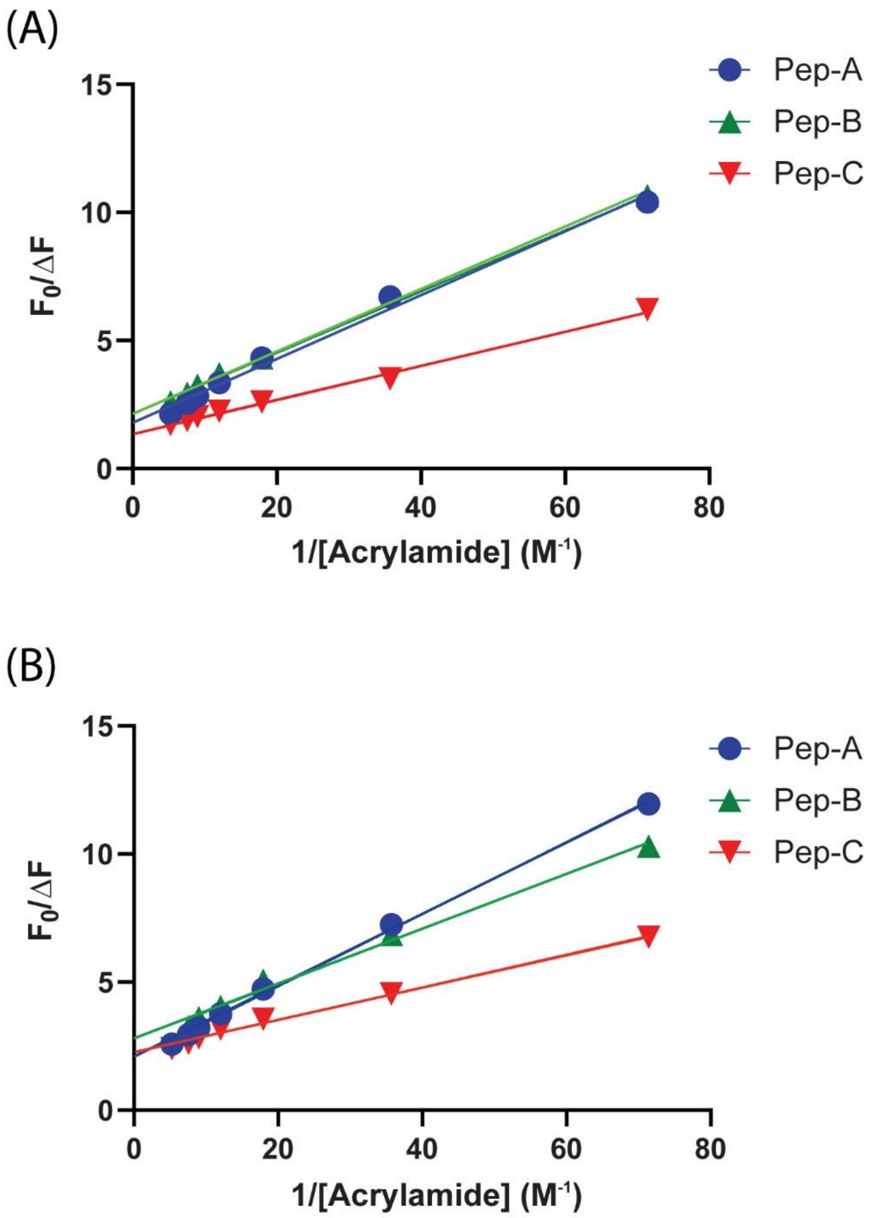

Figure S3. Stern Volmer Modified Plot for Acrylamide quenching of non-myristoylated peptides in presence of (A) POPC/POPG and (B) POPC vesicles.

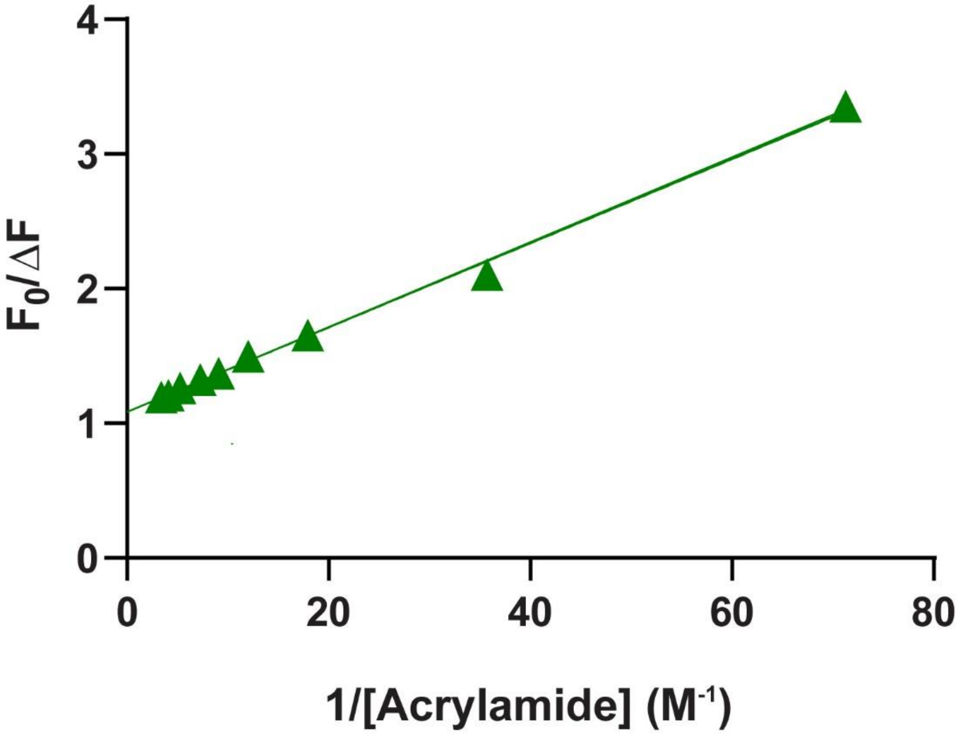

Figure S4. Stern Volmer Modified Plot for Acrylamide quenching of Myr-B in buffer.

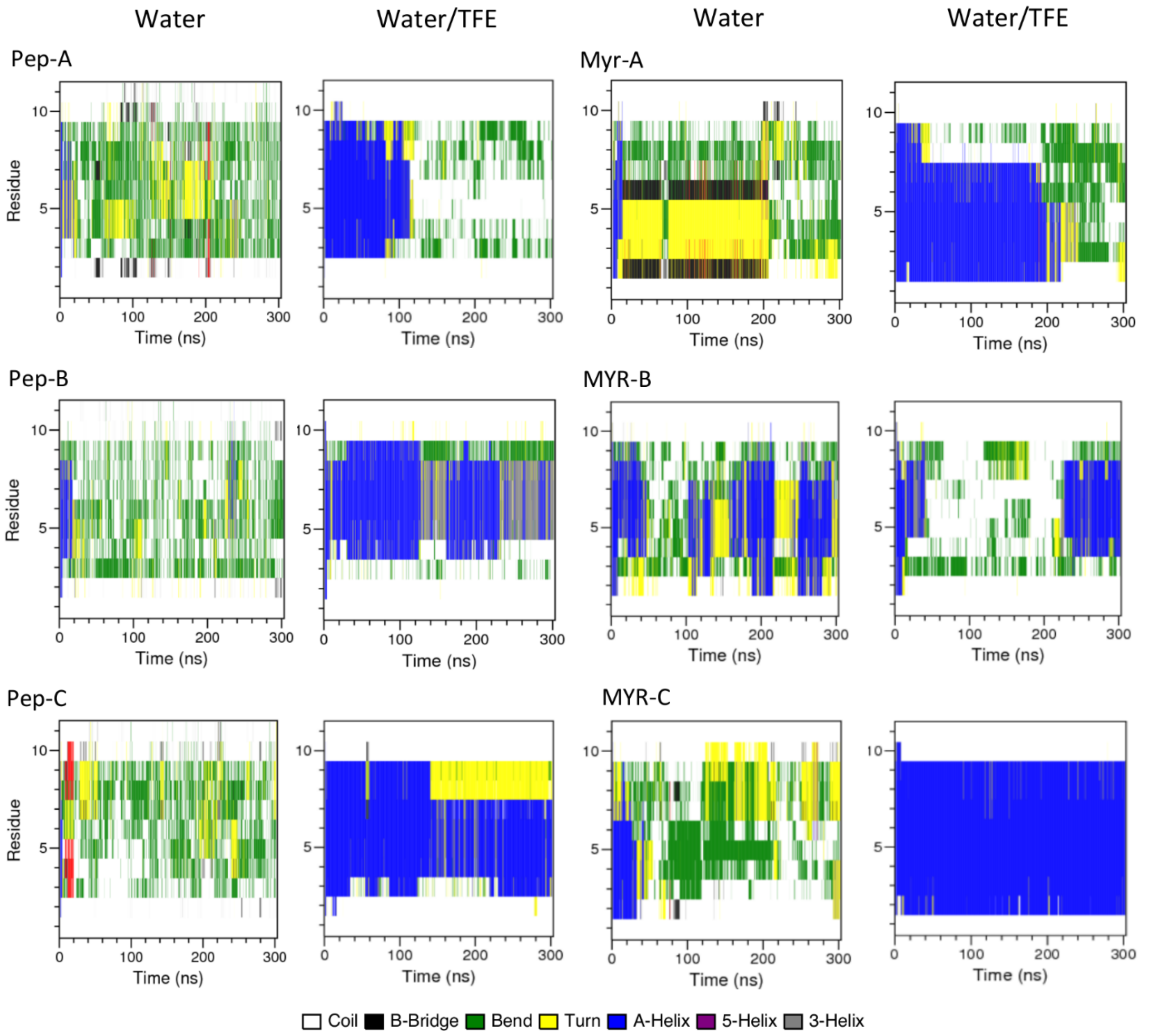

Figure S5. Time evolution of secondary structure of non-myristoylated and myristoylated peptides in water and TFE/water (50 % v/v).

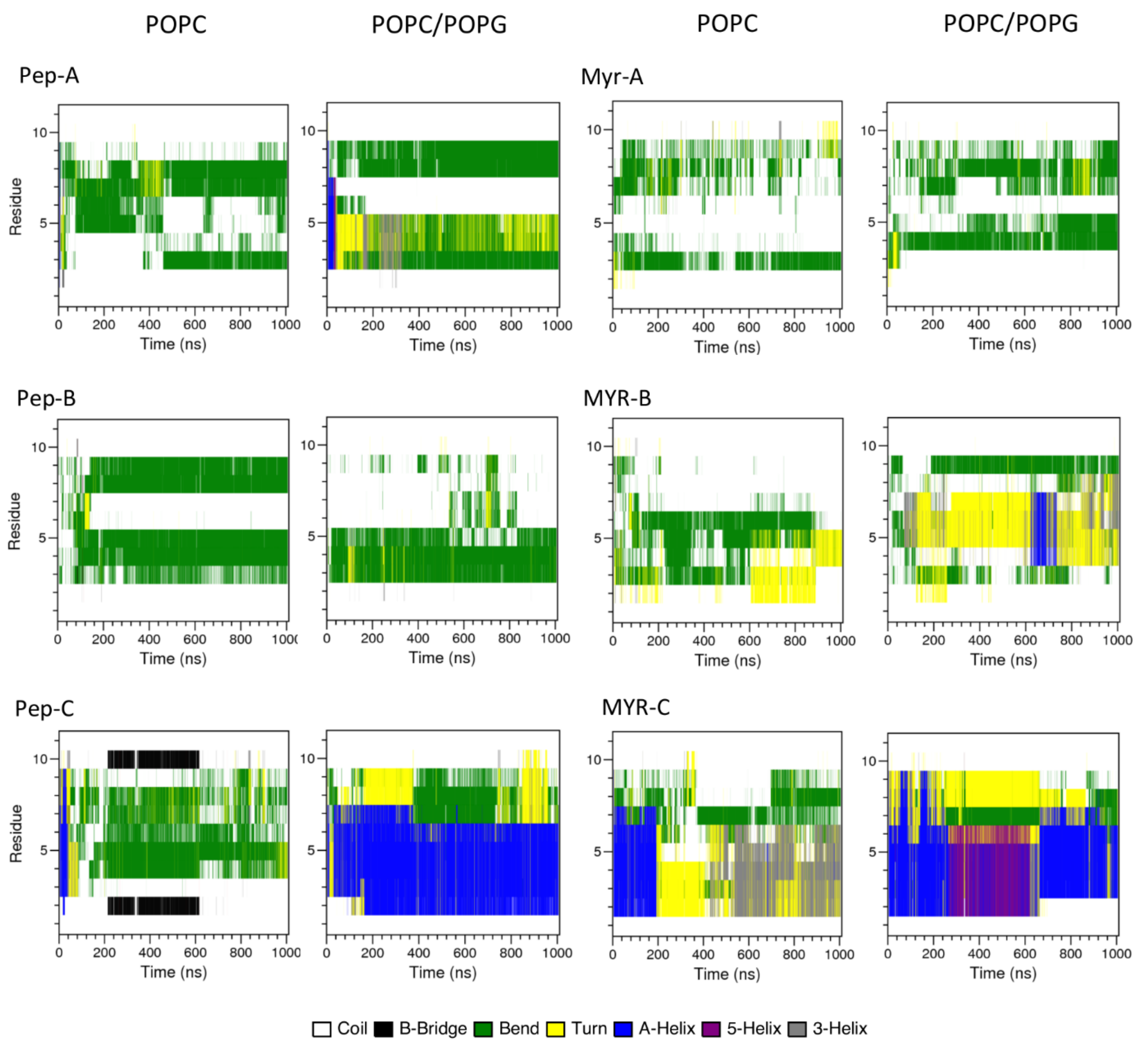

Figure S6. Time evolution of secondary structure of non-myristoylated and and myristoylated peptides in POPC and POPC/POPG (70/30 w/w) lipid membranes.

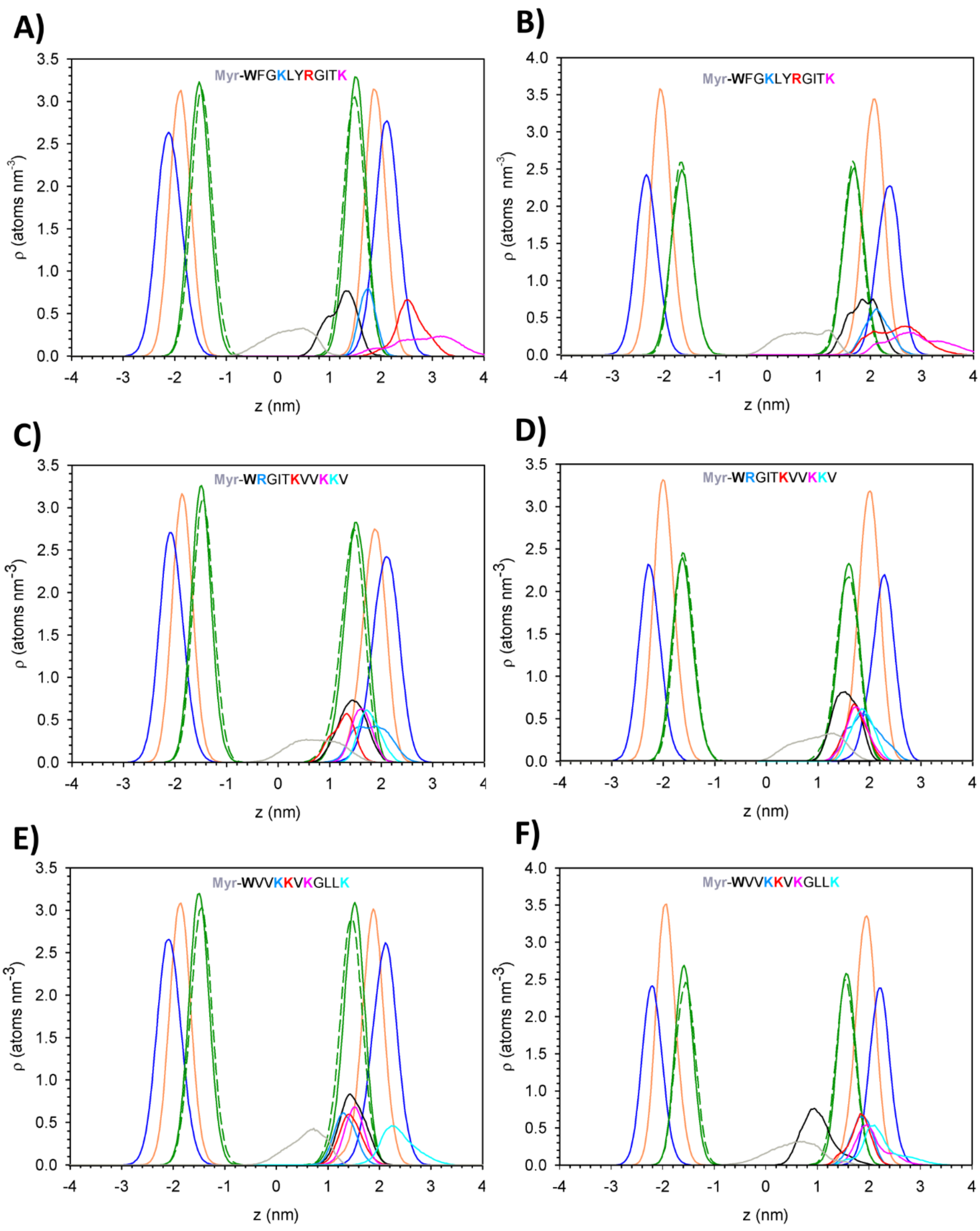

Figure S7. Average density profiles across lipid bilayer of phosphate (orange), nitrogen atoms of choline (blue), carbonyl atoms (green) of lipid molecules and myristoyl chain, Trp-1 and charged aminoacids (Lys and Arg) of (A) Myr-A in POPC, (B) Myr-A in POPC/POPG, (C) Myr-B in POPC, (D) Myr-B in POPC/POPG, (E) Myr-C in POPC and (F) Myr-C in POPC/POPG. The density is calculated with respect to the lipid bilayer center (z = 0).

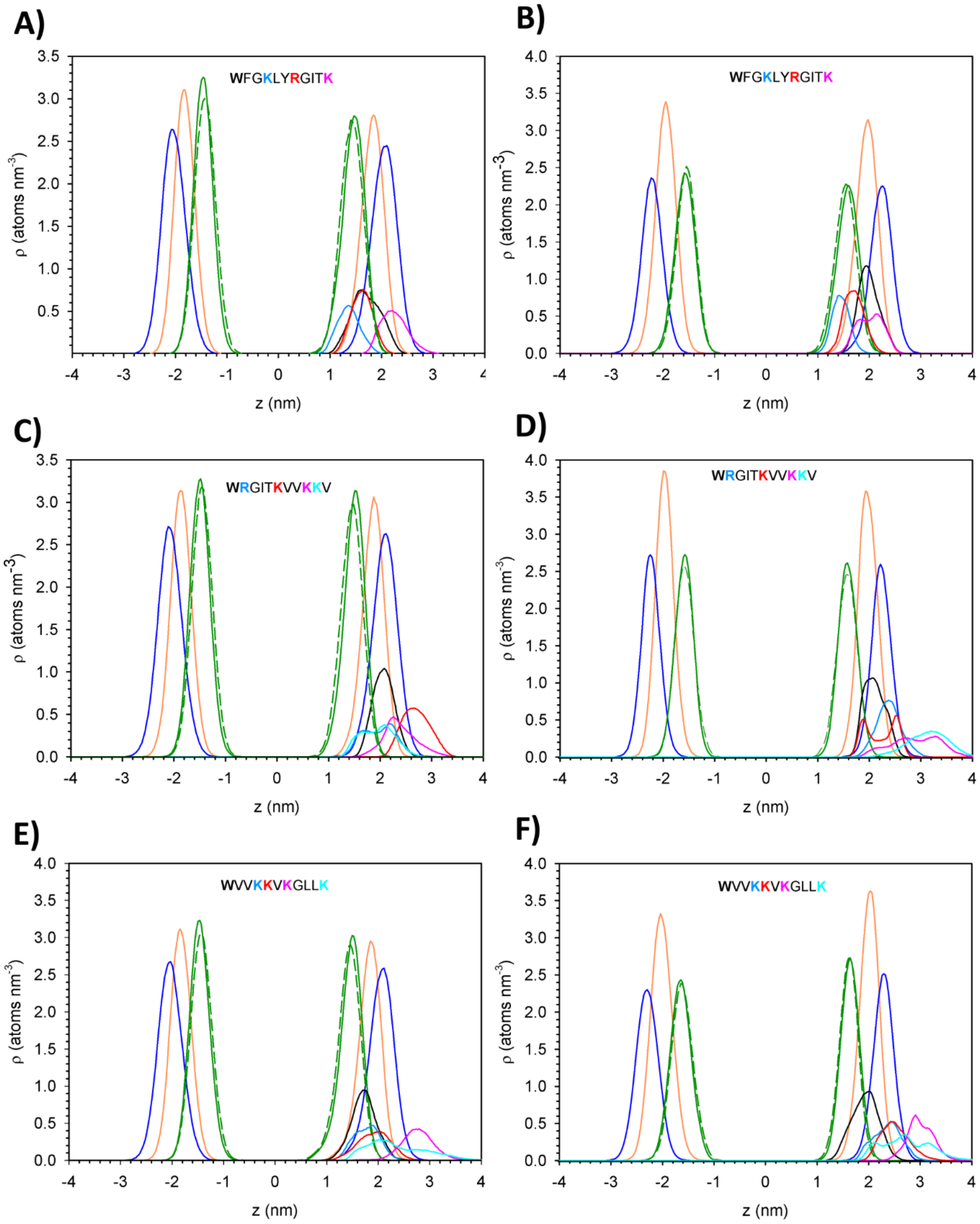

Figure S8. Average density profiles across lipid bilayer of phosphate (orange), nitrogen atoms of choline (blue), carbonyl atoms (green) of lipid molecules, Trp-1 and charged amino acids (Lys and Arg) of (A) Pep-A in POPC, (B) Pep-A in POPC/POPG, (C) Pep-B in POPC, (D) Pep-B in POPC/POPG, (E) Pep-C in POPC and (F) Pep-C in POPC/POPG. The density is calculated with respect to the lipid bilayer center (z = 0).

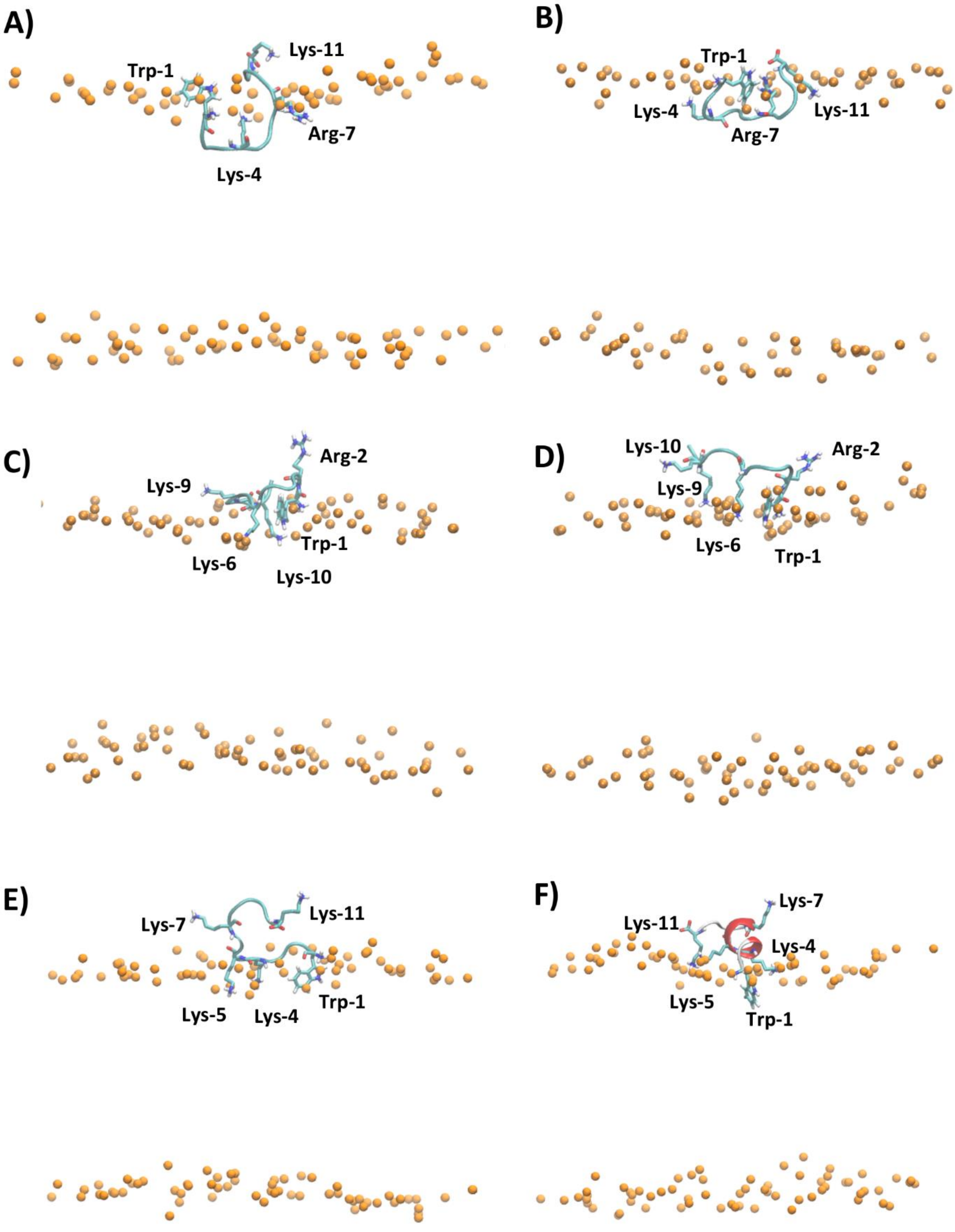

Figure S9. Snapshot of MD simulations at 1 μs of (A) Pep-A in POPC, (B) Pep-A in POPC/POPG, (C) Pep-B in POPC, (D) Pep-B in POPC/POPG, (E) Pep-C in POPC and (F) Pep-C in POPC/POPG. The phosphorous atoms of lipids are represented as orange dots. The residue of lysine, arginine and tryptophan are represented in stick whereas the hydrophobic amino acids are represented as orange stick. The water and counterions are omitted for clarity

Table S1. MIC values (mg/L) of different myristoylated and non-myristoylated peptides against 10 different isolates of *Candida albicans*, *Candida glabrata, Candida parapsilosis, Candida tropicalis* and *Candida Auris.*

Candida spp.	Pep-A	**Myr-A**	Pep-B	**Myr-B**	Pep-C	**Myr-C**
Candida albicans 1	>256	16	>256	16	>256	32
Candida albicans 2	>256	16	>256	16	>256	32
Candida albicans 3	>256	16	>256	16	>256	32
Candida albicans 4	>256	16	>256	16	>256	32
Candida albicans 5	>256	8	>256	16	>256	32
Candida albicans 6	>256	16	>256	16	>256	32
Candida albicans 7	>256	16	>256	16	>256	32
Candida albicans 8	>256	16	>256	16	>256	32
Candida albicans 9	>256	16	>256	16	>256	32
Candida albicans 10	>256	16	>256	16	>256	32
Candida glabrata 1	>256	16	>256	32	>256	32
Candida glabrata 2	>256	16	>256	32	>256	32
Candida glabrata 3	>256	16	>256	32	>256	32
Candida glabrata 4	>256	16	>256	32	>256	32
Candida glabrata 5	>256	16	>256	16	>256	32
Candida glabrata 6	>256	16	>256	32	>256	32
Candida glabrata 7	>256	16	>256	32	>256	32
Candida glabrata 8	>256	16	>256	32	>256	32
Candida glabrata 9	>256	16	>256	32	>256	32
Candida glabrata 10	>256	16	>256	32	>256	32
Candida parapsilosis 1	>256	16	>256	32	>256	32
Candida parapsilosis 2	>256	16	>256	32	>256	32
Candida parapsilosis 3	>256	16	>256	32	>256	32
Candida parapsilosis 4	>256	16	>256	32	>256	32
Candida parapsilosis 5	>256	16	>256	32	>256	32
Candida parapsilosis 6	>256	16	>256	32	>256	32
Candida parapsilosis 7	>256	16	>256	32	>256	32

Candida parapsilosis 8	>256	16	>256	32	>256	32
Candida parapsilosis 9	>256	16	>256	32	>256	32
Candida parapsilosis 10	>256	16	>256	32	>256	32
Candida tropicalis 1	>256	16	>256	8	>256	16
Candida tropicalis 2	>256	16	>256	8	>256	16
Candida tropicalis 3	>256	16	>256	8	>256	16
Candida tropicalis 4	>256	16	>256	8	>256	16
Candida tropicalis 5	>256	16	>256	8	>256	16
Candida tropicalis 6	>256	16	>256	8	>256	16
Candida tropicalis 7	>256	16	>256	8	>256	16
Candida tropicalis 8	>256	16	>256	8	>256	16
Candida tropicalis 9	>256	16	>256	8	>256	16
Candida tropicalis 10	>256	16	>256	8	>256	16
Candida Auris 1	>256	>256	>256	8	>256	16
Candida Auris 2	>256	>256	>256	8	>256	16
Candida Auris 3	>256	>256	>256	16	>256	32
Candida Auris 4	>256	>256	>256	32	>256	32
Candida Auris 5	>256	>256	>256	32	>256	64
Candida Auris 6	>256	>256	>256	16	>256	32
Candida Auris 7	>256	>256	>256	16	>256	32
Candida Auris 8	>256	>256	>256	16	>256	32
Candida Auris 9	>256	>256	>256	32	>256	16
Candida Auris 10	>256	>256	>256	32	>256	32
